# Enhancing performance of a miniaturized surface plasmon resonance sensor in the reflectance detection mode using a waveguide-coupled bimetallic chip

**DOI:** 10.1186/1556-276X-8-344

**Published:** 2013-08-06

**Authors:** Yeon Kyung Lee, Dae Ho Jang, Kyeong-Seok Lee, Won Mok Kim, Young-Soo Sohn

**Affiliations:** 1 Department of Biomedical Engineering, Catholic University of Daegu, 13-13 Hayang-ro, Hayang-eup, Gyeongsan-si, Gyeongbuk 712-702, Republic of Korea; 2 KDmedia, Inc. Bio Laboratory, SungKyunKwan University, 2066 Seobu-ro, Jangan-gu, Suwon 440-746, Republic of Korea; 3 Electronic Material Research Center, Korean Institute of Science and Technology, 14-5 Hwarang-ro, Seongbuk-gu, Seoul 136-791, Republic of Korea

**Keywords:** Waveguide-coupled bimetallic chip, Miniaturized SPR sensor, Intensity measurement, Low molecular weight biomolecule

## Abstract

The characteristics of a waveguide-coupled bimetallic (WcBiM) chip in a miniaturized surface plasmon resonance (SPR) sensor and its detection capability for a low molecular weight biomolecule were investigated. The configuration of the WcBiM chip was gold (Au)/waveguide (ZnS-SiO_2_)/silver (Ag). In the intensity measurement mode, the sensitivity could be improved by reducing the full width at half maximum (FWHM) of the reflectance curve. The FWHM of the WcBiM chip is narrower than that of the Au chip, which suggests that the slope of the reflectance curve for the WcBiM chip is steeper. In order to generate enhanced resolution, the reflectance should be monitored at the specific angle where the slope is the steepest in the reflectance curve. For the detection of biotin that is a low molecular weight biomolecule, streptavidin was formed on the SPR sensor chip surface. The response of the SPR to biotin at various concentrations was then acquired. The sensitivities of the WcBiM chip and the Au chip were 0.0052%/(ng/ml) and 0.0021%/(ng/ml), respectively. The limit of detection of the biotin concentration for both the WcBiM and Au chips was calculated. The values were 2.87 ng/ml for the WcBiM chip and 16.63 ng/ml for the Au chip. Enhancement of the sensitivity in the intensity detection mode was achieved using the WcBiM chip compared with the Au chip. Therefore, sufficient sensitivity for the detection of a disease-related biomarker is attainable with the WcBiM chip in the intensity measurement mode using a miniaturized SPR sensor.

## Background

With continuous research and advancement over the last several decades, a surface plasmon resonance (SPR) sensor has been developed as a promising technology for biomolecular interaction analysis (e.g., antigen-antibody reaction, DNA) due to its merits of real-time monitoring and higher sensitivity compared with any other sensor system [1-3]. In addition, an SPR sensor does not require any chemical procedures such as fluorescence. Thus, this sensor has been studied for the detection of disease-related biomarkers, which requires immediate detection and simple operation [4,5]. The SPR sensor is based on variations in permittivity, such as the refractive index on a metal surface, and is very sensitive to subtle changes. When a small amount of the target analyte binds with the bioreceptors immobilized on the metal surface, the reflectance curve, acquired by monitoring the reflected light intensity on changing the incident angle of the light source, shifts depending on the changed refractive index of the bound target biomolecule. Based on these principles, various diseases can be diagnosed by detecting disease-related biomarkers [6,7].

The SPR-based sensor relies on the extraordinary optical properties of noble metals such as gold (Au), silver (Ag), aluminum (Al), and copper (Cu) [8]. Among these metals, Au has been commonly used as an SPR sensor chip since it has merits of great stability, durability, and outstanding biocompatibility [8-10]. Although a single Au layer leads to stable performance, the commercialized Au-based sensor chip has a sensitivity limitation when it comes to the detection of biomolecules with very low molecular weight or trace level concentration [11]. The detection ability of biomolecules at trace level concentration or very low molecular weight plays an important role in the instrument for the early diagnosis of diseases. The SPR sensor utilizes the evanescent field, which measures changes in the refractive index in proximity to the metal surface [12]. Compared to Au, Ag enhanced an evanescent field better, resulting in a sharper SPR reflectance curve [13,14]. However, Ag is easily oxidized when exposed to an air or liquid environment due to its high oxygen affinity [13,15]. As a remedy for the shortcomings of the Au and Ag sensor chips, the Ag-Au bimetallic SPR chip has been proposed to exploit their advantages [9,16]. Commonly, the thin Au film is coated over the surface of the Ag film due to the chemical stability of the Au metal [14]. In addition, the waveguide layer has been adopted to obtain a sharper reflectance curve and moderate decay length [17]. As materials for the waveguide layer, Si_3_N_4_[18], SiO_2_[19], and ZnO [20] have been extensively studied.

There are four different SPR detection modes, which indicate angular interrogation, intensity measurement, phase interrogation, and wavelength measurement [19,21]. Among these approaches, the angular and intensity detection schemes have been widely used as the SPR measurement mode. Angular interrogation [22] detects the SPR angle change by monitoring the SPR reflectance dip shift. This offers highly sensitive performance by measuring extremely small angle changes of the SPR using the Au chip with a broad SPR reflectance curve. The intensity measurement [23] monitors the intensity of the reflected light at a fixed angle where the maximum slope of the SPR reflectance curve is located. This method is very effective in the case of an SPR reflectance curve with a narrower full width at half maximum (FWHM), leading to great reflectance variation at this fixed angle [24,25].

In the present work, we experimentally investigated the characteristics of a waveguide-coupled bimetallic (WcBiM) chip in the intensity measurement mode using the miniaturized SPR sensor system, and extended the study to the system sensitivity for the detection of biotin with very low molecular weight (MW 341.38) at a low concentration level. The noble metal materials applied to the WcBiM chip were Ag as the inner metal layer and Au as the outer metal layer. Moreover, ZnS-SiO_2_ was used as a waveguide layer due to the high force of adhesion between the two metals. It is easy and robust to integrate this waveguide layer with electrical and optical systems [18]. The characteristics of the WcBiM chip in the intensity measurement were investigated by evaluating the FWHM and slope of the SPR reflectance curve. The comparison analysis of streptavidin-biotin interaction was carried out using a miniaturized SPR sensor in the intensity measurement with both the WcBiM and Au chips.

## Methods

### Surface plasmon resonance sensor system

**Figure 1:**
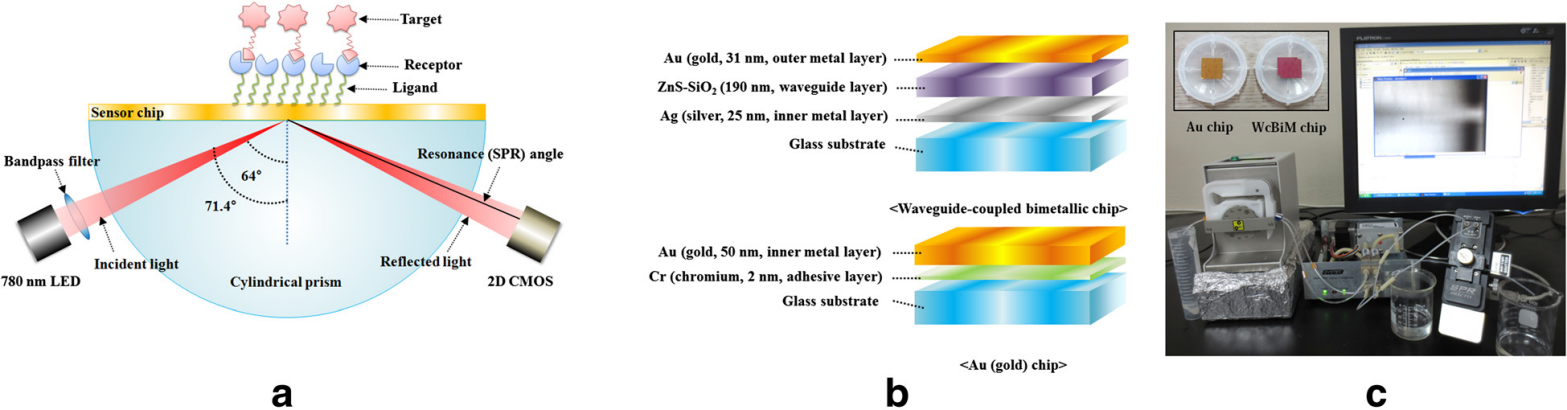
**Schematic diagram of an SPR sensor, configuration of the chips, and experimental setup. (a)** A schematic diagram of a miniaturized SPR sensor system, **(b)** the configuration of the WcBiM chip and the conventional Au chip, and **(c)** experimental setup and both the fabricated WcBiM and Au sensor chips.

A schematic diagram of a simple and miniaturized SPR sensor system is depicted in Figure 1a. The SPR size was 45 mm × 140 mm × 130 mm. The p-polarized beam from a 780-nm light-emitting diode (LED) passed through a band-pass interference filter (780 ± 5 nm) and was directed to the SPR sensor chip through a cylindrical prism (BK7). Then, the intensity of the reflected light beam was monitored using a two-dimensional complementary metal oxide semiconductor (2D-CMOS) with an image acquisition board. The incident beam angle range was 64.0° to 71.4°. The fluidic module of the SPR sensor system has two channels: a sample channel for analyte injection and a reference channel for reference solution injection. The reason for this is that this SPR sensor system does not have a thermostat and can be affected by outer environmental factors such as temperature. Thus, a meaningful SPR signal for analyzing the biomolecular interactions was obtained by subtracting the reference signal from the sample signal. All solutions were circulated through the flow cell of the SPR sensor at 20 μl/min of flow rate using a peristaltic tubing pump. The degasser was used to remove air bubbles before the samples were placed in the SPR sensor.

### Waveguide-coupled bimetallic chip

The configuration of the WcBiM SPR chip is shown in Figure 1b. This was prepared by the deposition of gold (Au), waveguide (ZnS-SiO_2_), and silver (Ag) onto the glass substrate using an RF magnetron. The thickness of each layer was Au (31 nm)/ZnS-SiO_2_ (190 nm)/Ag (25 nm), which was optimized using a commercial optical thin film software (SCI Film Wizard™, Carlsbad, CA, USA). ZnS-SiO_2_ was adopted as a waveguide because it exhibits a good adhesion property between Ag and Au. For verification of the performance of the WcBiM chip, it was compared with the commercialized Au chip (K-MAC, Daejeon, Korea). The Au chip consists of Au (50 nm)/Cr (2 nm) on a glass substrate. Experimental setup is represented in Figure 1c, and both WcBiM and Au chips are shown in the inset of Figure 1c.

### Materials and detection of biotin

Streptavidin (Sigma-Aldrich, St. Louis, MO, USA) was immobilized on the sensor chip modified by a self-assembled monolayer (SAM; K-MAC, Daejeon, Korea) containing *N*-hydroxysuccinimide and ethyl(dimethylaminopropyl) carbodiimide so that the amine group would react easily. The WcBiM SPR chip was dipped in 1 mM SAM solution in ethanol (2.5 ml) overnight. The streptavidin molecules were covalently immobilized onto the sensor chip by injection of the streptavidin solution into the sensor system. Next, the biotin (Sigma-Aldrich, St. Louis, MO, USA) was made to flow into the SPR sensor system in order of concentration at 50, 100, 150, and 200 ng/ml. All proteins were diluted in the phosphate-buffered saline (Sigma-Aldrich, St. Louis, MO, USA) solution.

## Results and discussion

**Figure 2:**
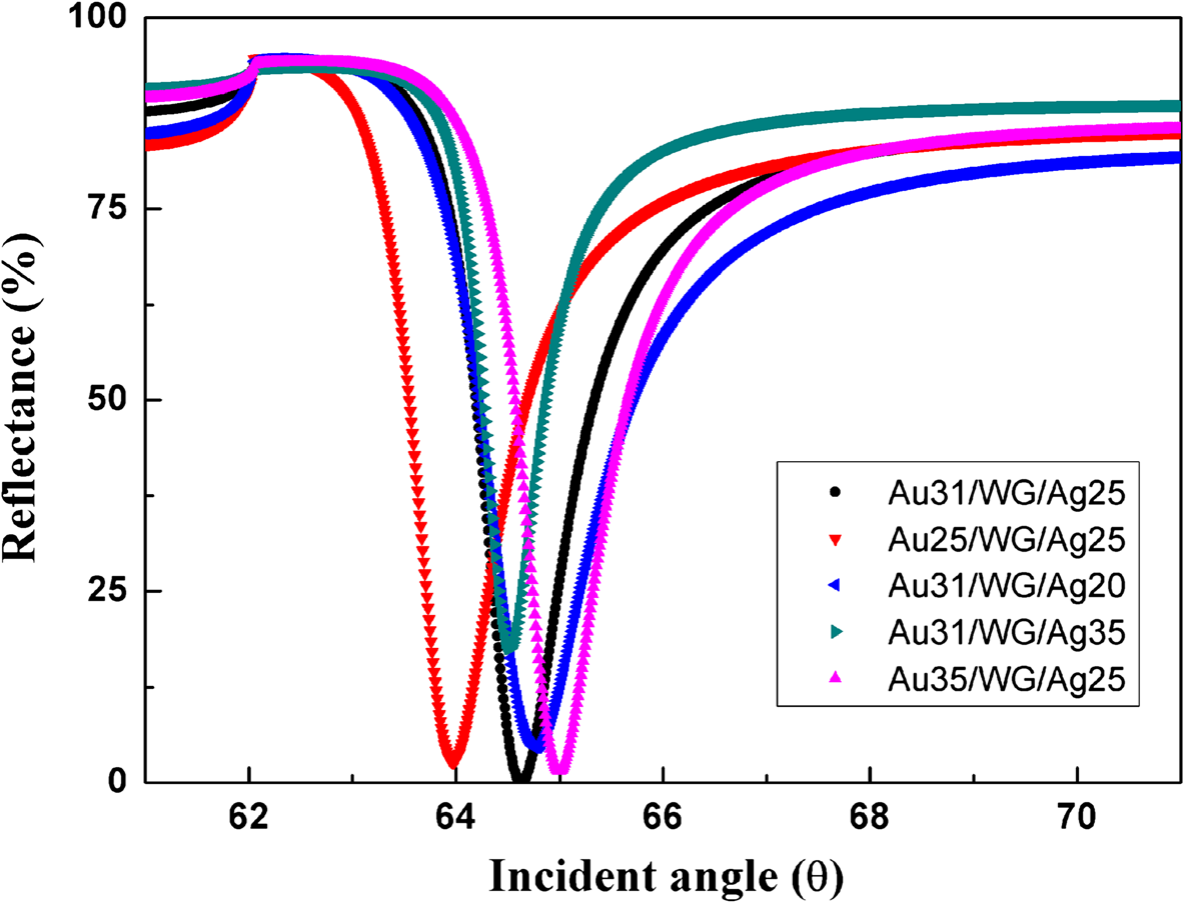
Reflectance curves of the five different WcBiM configurations.

In order to get the optimal configuration, the sensing characteristics of five different configurations of the WcBiM SPR chips were investigated and compared using the commercial optical thin film software (SCI Film Wizard™) as shown in Figure 2. The five configurations were Au (31 nm)/ZnS-SiO_2_ (190 nm)/Ag (25 nm), Au (25 nm)/ZnS-SiO_2_ (190 nm)/Ag (25 nm), Au (31 nm)/ZnS-SiO_2_ (190 nm)/Ag (20 nm), Au (31 nm)/ZnS-SiO_2_ (190 nm)/Ag (35 nm), and Au (35 nm)/ZnS-SiO_2_ (190 nm)/Ag (25 nm). The thickness of the waveguide was fixed. In this calculation, the refractive indices of the BK7 and PBS were set to be 1.515 and 1.335, respectively. The line widths of the reflectance curve for each stack were close to each other. When biomolecules are adsorbed onto the sensor chip, then the refractive index is changed. Thus, we assumed that the refractive index was changed from 1.335 to 1.35, and the change in the reflectance was calculated at the angle where the steepest slope is. The largest change in the reflectance among the five configurations occurs at the Au (31 nm)/ZnS-SiO_2_ (190 nm)/Ag (25 nm) and Au (35 nm)/ZnS-SiO_2_ (190 nm)/Ag (25 nm) chips as listed in Table 1. However, the Au (31 nm)/ZnS-SiO_2_ (190 nm)/Ag (25 nm)-configured chip has the deepest dip depth (minimum reflectance = 0.005%) in the reflectance curve compared to the other configuration (minimum reflectance = 1.507%). This deepest dip depth may lead to a larger dynamic range in the sensor application.

**Table 1:** SPR parameters for WcBiM configurations when the refractive index is changed from 1.335 to 1.35

**Configuration**	**Minimum reflectance**	**Resonance angle**	**Steepest slope**	**Reflectance at *n *= 1.335**	**Reflectance at *n *= 1.35**	**Δ*R***
						**(*R***_***n *= 1.35**_**− *R***_***n *= 1.335**_**)**
	**(%)**	**(deg)**	**(Δ *R */Δ *θ *)**	**(%)**	**(%)**	**(%)**
Au(31 nm)/WG/Ag(25 nm)	0.005	64.63	−155.8	29.86	92.82	62.96
Au(25 nm)/WG/Ag(25 nm)	2.697	63.97	−156.0	33.51	93.78	60.27
Au(31 nm)/WG/Ag(20 nm)	4.608	64.77	−115.8	33.69	91.83	58.14
Au(31 nm)/WG/Ag(35 nm)	17.528	64.51	−181.7	39.97	93.03	53.06
Au(35 nm)/WG/Ag(25 nm)	1.507	65.00	−154.3	29.50	92.46	62.96

*WG* waveguide.

**Figure 3:**
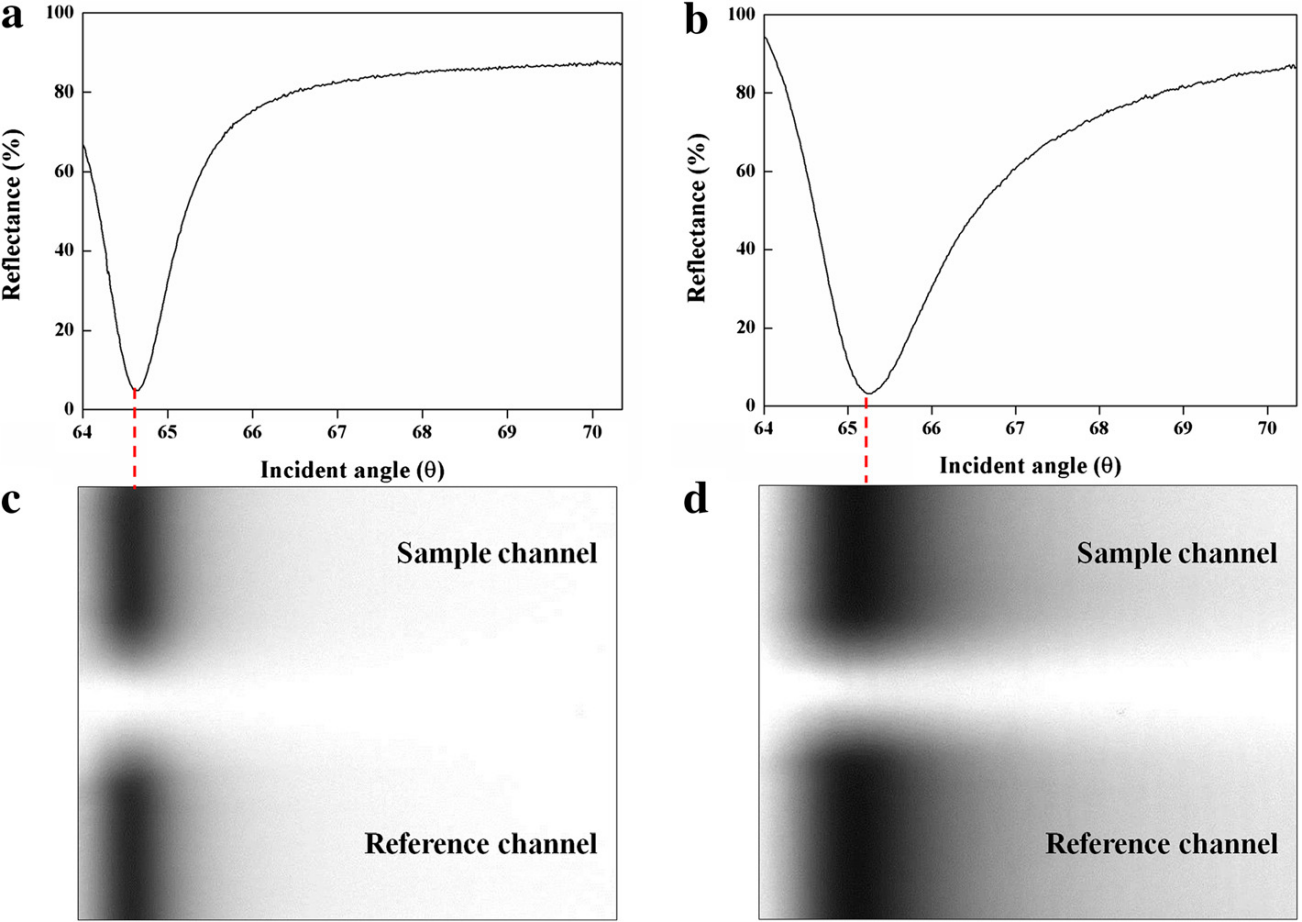
Reflectance curves (a, b) corresponding grayscale images (c, d) for the WcBiM and Au chips, respectively.

For the analysis for the biomolecular interactions using the WcBiM chip and the Au chip, the SPR reflectance curves were first obtained. The grayscale images and their corresponding reflectance curves are shown in Figure 3a,b,c,d. The dark portion in the image signifies that there was negligible reflected light intensity, which corresponds to the reflectance dip. Such intensity profiles for a dual channel are commonly used to demonstrate the proper alignment of the SPR system. The upper and lower grayscale intensity profiles in Figure 3c,d correspond to the reflectance of the sample and reference channels, respectively. The images revealed that the WcBiM chip had a narrower dark area than the Au chip. The SPR reflectance curve data points were plotted as solid lines in Figure 3a,b by successive numerical fitting of the intensity profiles generated from the SPR. As shown in Figure 3a,b, the resonance angles that had minimum reflectance for the WcBiM and Au chips were 64.64° with 4.83% and 65.26° with 3.22%, respectively. The FWHM of the WcBiM SPR chip was narrower than that of the commercialized Au SPR chip, and the FWHMs of the WcBiM chip and the Au chip were 0.94° and 1.89°, respectively. Thus, among the four different detection modes - angular interrogation, intensity measurement, phase interrogation, and wavelength measurement - the WcBiM SPR chip can be utilized to improve the resolution in the intensity measurement mode since it has a sharper reflectance curve [19].

**Figure 4:**
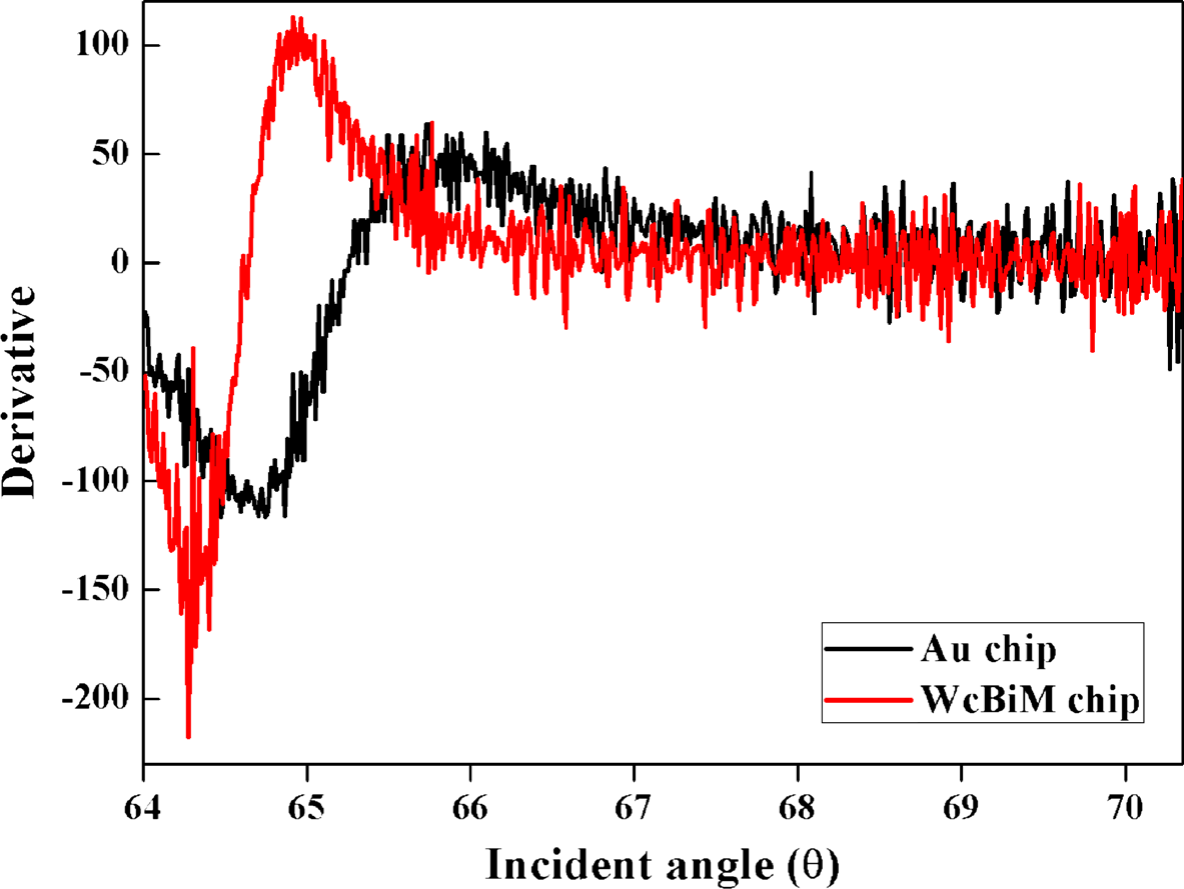
Derivative with respect to the incident angle for both the WcBiM and Au chips.

In order to achieve a better resolution, it is wise to monitor the reflectance at the specific pixel of the 2D-CMOS that corresponds to the angle where the slope is the steepest in the reflectance curve. Therefore, the reflectance curve was differentiated with respect to the incident angle, as shown in Figure 4, and the absolute value of these derivatives indicating the gradient of the SPR reflectance curve was obtained. As a result, the steepest slopes in the reflectance curves for the WcBiM chip and the Au chip were −237.52%/° at 64.28° and −115.92%/° at 64.86°, respectively. Thus, the WcBiM chip had a gradient that was two times steeper in the SPR reflectance curve than the Au chip. From these results, the sensitivity of the WcBiM chip can be expected to be higher than that of the Au chip.

**Figure 5:**
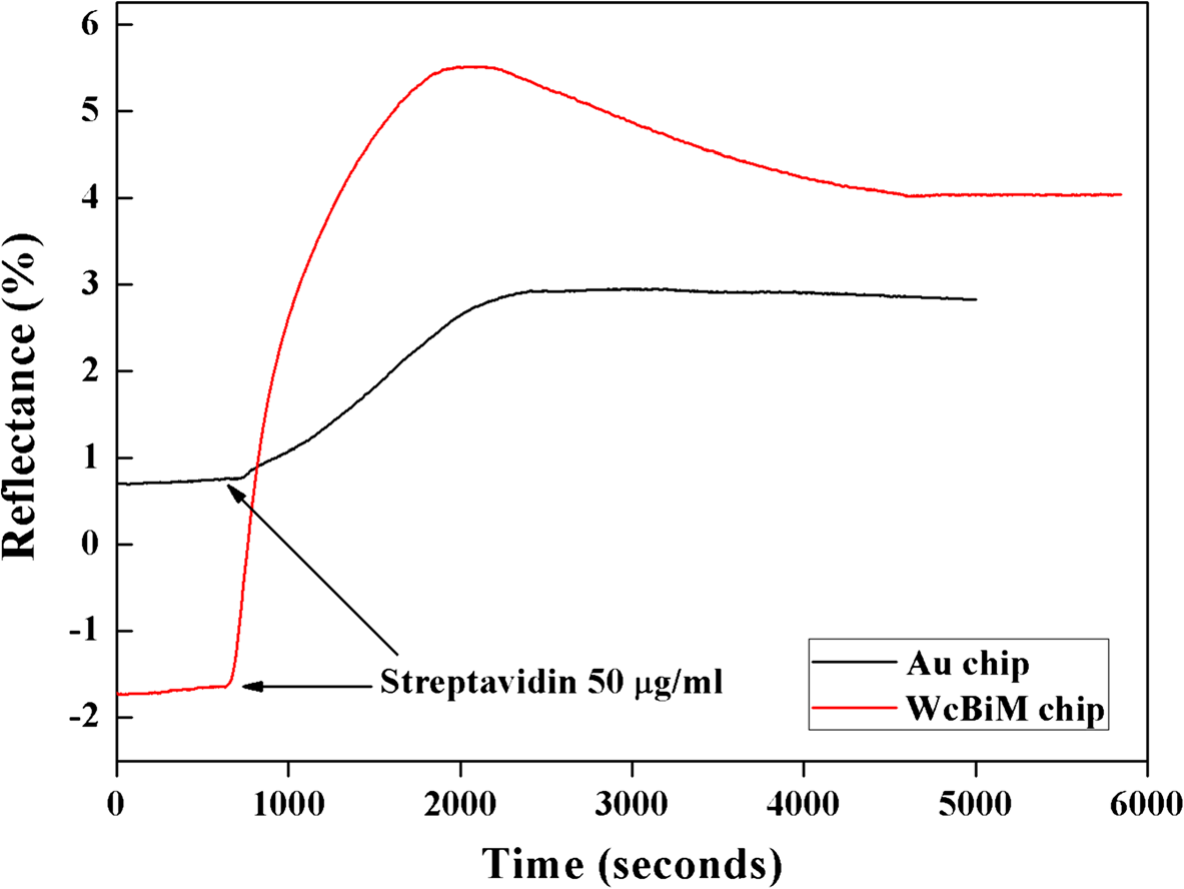
SPR responses to the streptavidin for the WcBiM chip and the Au chip.

**Figure 6:**
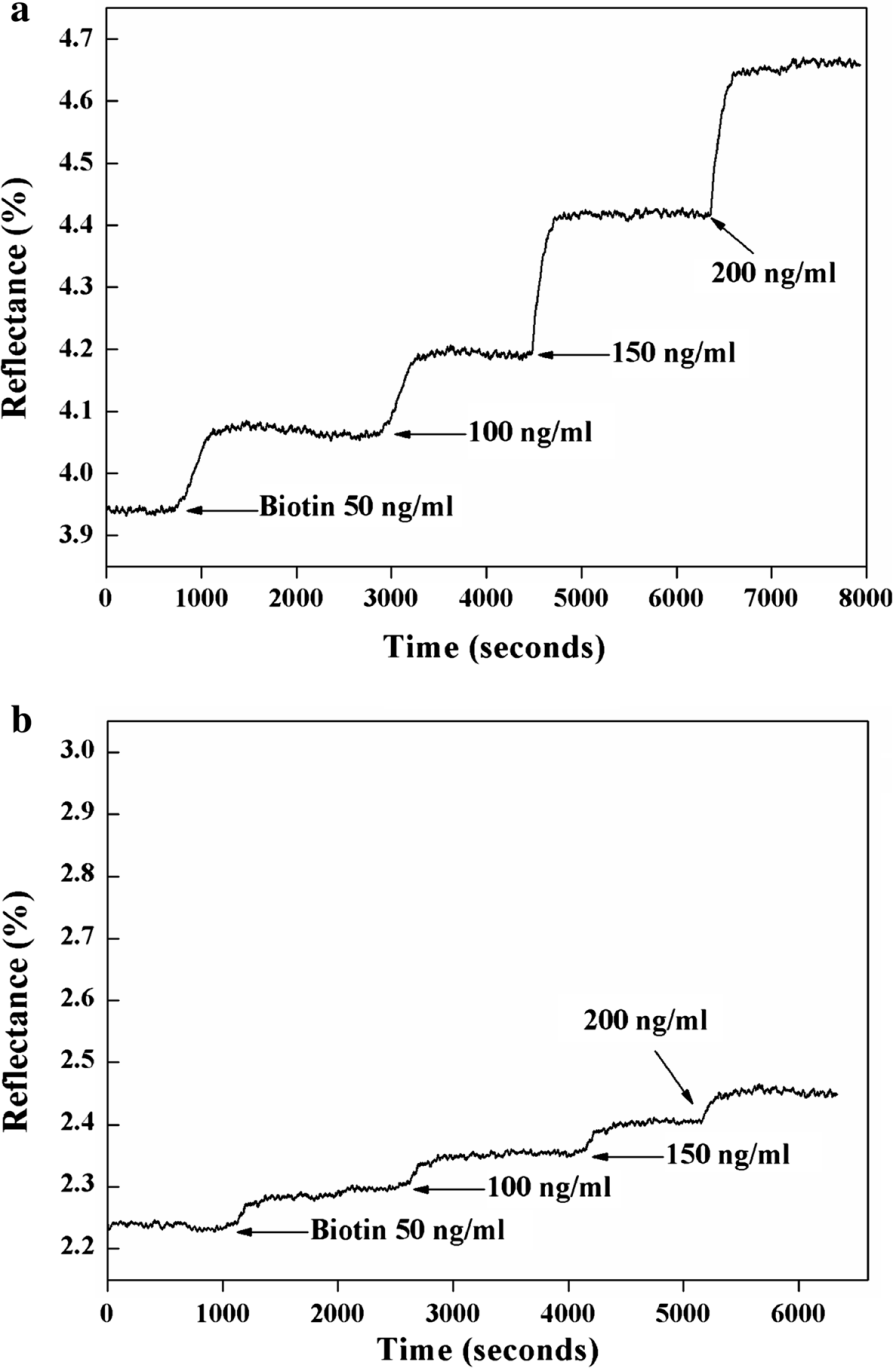
**SPR responses to biotin for (a) the WcBiM chip and (b) the Au chip.** The biotin has concentrations ranging from 50 to 200 ng/ml.

For verification of the detection capability of the WcBiM chip, a dynamic experiment was carried out with streptavidin-biotin interaction. The streptavidin-biotin interaction led to a shift in the resonance angle, and the change in the reflectance was monitored at the specified angle. Streptavidin, with relatively larger molecular weight, has four binding sites that can react to biotin, with very low molecular weight; streptavidin has a very high affinity with biotin. If biomolecules with very low molecular weight such as biotin can be detected with high sensitivity, it is very useful to detect a disease-related biomarker with low molecular weight or a trace level concentration. A 50-μg/ml concentration of streptavidin was formed on the SPR sensor chip surface, and biotin with various concentrations of 50, 100, 150, and 200 ng/ml was injected into the sensor surface to investigate the response. The SPR responses of the streptavidin for the WcBiM chip and the Au chip were 3.4349% and 1.3054%, respectively, as shown in Figure 5. The SPR response was obtained from the difference between the reflectance before the streptavidin injection and the reflectance after the streptavidin injection. We considered that the meaningful reflectance would be the mean value of the output signal for 100 s in the stable state. The average changes in the reflectance due to injection of the biotin with concentrations ranging from 50 to 200 ng/ml were 0.1360%, 0.3968%, 0.6524%, and 0.9141% for the WcBiM chip and 0.0415%, 0.1212%, 0.2213%, and 0.3347% for the Au chip for three replicates. The reflectance changes due to injection of the biotin with various concentrations of 50, 100, 150, and 200 ng/ml for an experiment for both the WcBiM and Au chips were shown in Figure 6a,b, respectively. This showed that the narrower the FWHM in the SPR reflectance for the WcBiM chip, the higher the corresponding SPR response.

**Figure 7:**
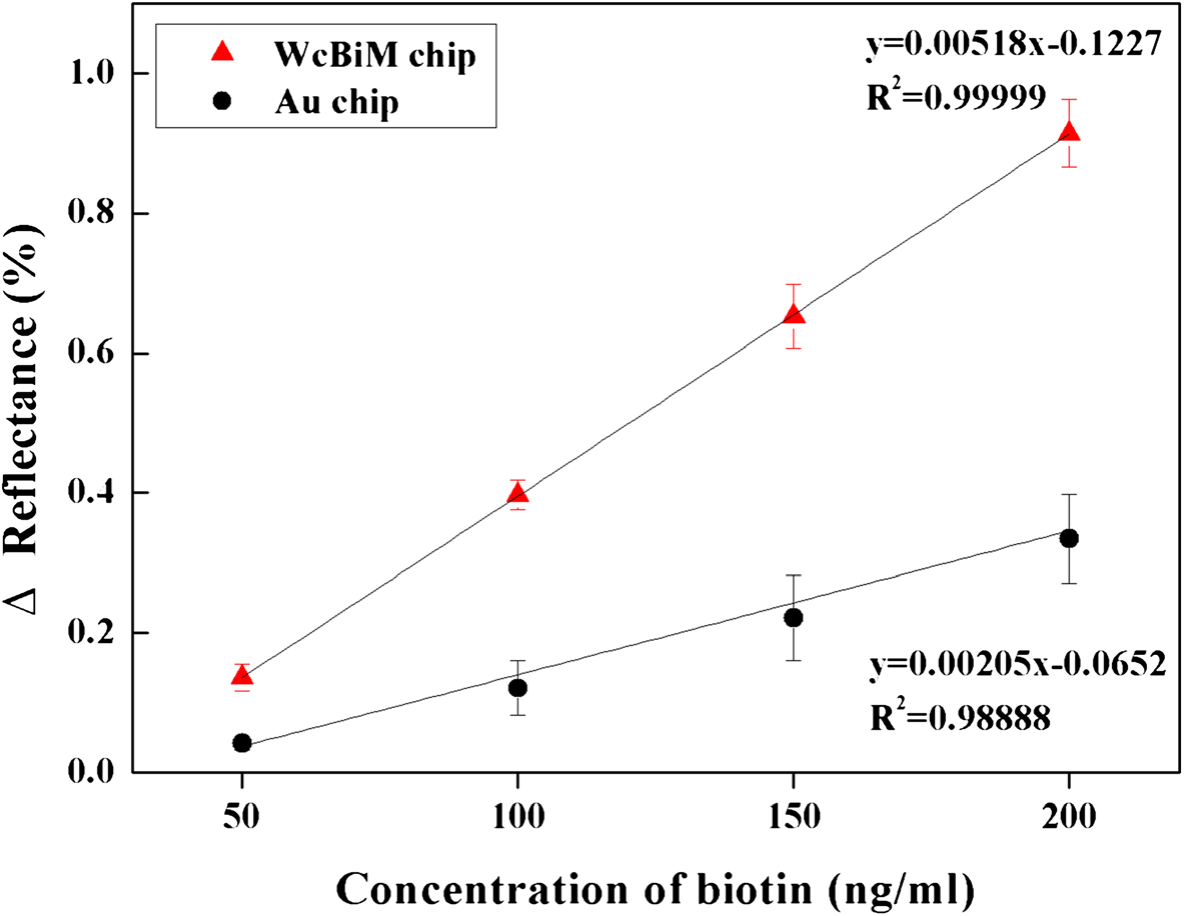
Linearity in calibration curves between SPR response and biotin concentration ranging from 50 to 200 ng/ml.

To confirm these results, Figure 7 presents the reflectance change as a function of the concentration of biotin. The sensitivity can be defined as the derivative of the SPR response with respect to the analyte concentration and is an extract from the linear part of the calibration curve for each of the investigated analytes. Thus, the sensitivity can be obtained by the slope (ΔReflectance (%)/ΔConcentration (ng/ml)) of their respective linear relations. The slopes for the SPR responses of biotin in the WcBiM chip and the Au chip were 0.0052%/(ng/ml) and 0.0021%/(ng/ml), respectively. This shows that the sensitivity of the WcBiM chip was twice that of the Au chip. Thus, the experimental results showed that the WcBiM chip enhances sensitivity in the reflectance measurement mode.

(1) $${\mathrm{Concentration}}_{\mathrm{LOD}}=\begin{array}{l} \frac{\mathrm{Concentration}\;\mathrm{of}\;\mathrm{the}\;\mathrm{analyte}}{\mathrm{SPR}\;\mathrm{response}}\\ \times 3\;\mathrm{Standard}\;\mathrm{deviation} \end{array}$$

The limit of detection (LOD) of this SPR sensor system was obtained using Equation 1 [26]. The standard deviation (SD) of the signal was recorded over 100 s in the stable state. The SDs of the blank measures for the WcBiM chip and the Au chip were 0.0026% and 0.0046%, respectively. Moreover, the SPR responses of 50 ng/ml biotin for both sensor chips were 0.1360% and 0.0415%, respectively. Therefore, the LOD of the concentration (concentration_LOD_) was calculated from Equation 1; the respective values were 2.87 ng/ml for the WcBiM chip and 16.63 ng/ml for the Au chip. Thus, the WcBiM chip can detect biomolecules at a very low level of concentration. From these results, if the SPR reflectance curve has a narrower FWHM and the detection mode is based on the intensity measurement, it is expected that the sensitivity of the sensor system can be enhanced compared with the conventional device. In particular, for the early diagnosis of diseases through the detection of a disease-related biomarker with very low molecular weight or trace level concentration, the SPR sensor in the reflectance detection mode using the WcBiM chip will be very useful tool for medical applications.

## Conclusions

The performance of a simplified SPR sensor with a WcBiM chip was investigated. Since the SPR sensor was simple and miniaturized, the incident angle of the beam was fixed. Thus, the reflectance curves for the designated incident angle were obtained by successive numerical fitting of the intensity profiles from 2D-CMOS for both WcBiM and Au chips. The FWHM of the Au chip was about twice as large as that of the WcBiM chip, which implied that the slope of the WcBiM reflectance curve was steeper. In order to achieve better performance, the reflectance was monitored at the specific pixel of the 2D-CMOS corresponding to the angle where the slope is the steepest in the reflectance curve. The slope was obtained by differentiating the reflectance curve with respect to the incident angle. The steepest slopes for the WcBiM chip and the Au chip were −237.52%/° and −115.92%/°, respectively. The WcBiM chip's slope was about twice as steep as that of the Au chip. For the detection of a disease-related biomarker, it is necessary for biomolecules with very low molecular weight such as biotin to be detected. For the verification of the detection capability of the WcBiM chip, an experiment of the streptavidin-biotin interaction was carried out. Streptavidin at a concentration of 50 μg/ml formed on the SPR sensor chip surface, and the response of the SPR to the biotin with various concentrations of 50, 100, 150, and 200 ng/ml was acquired in triplicate. The sensitivities of the WcBiM chip and the Au chip were 0.0052%/(ng/ml) and 0.0021%/(ng/ml), respectively. In addition, the concentration_LOD_ of this SPR sensor system was calculated. The results were 2.87 ng/ml for the WcBiM chip and 16.63 ng/ml for the Au chip. Thus, for the detection of a disease-related biomarker, an SPR sensor in the reflectance detection mode using the WcBiM chip would be very useful in the medical field.

## Competing interests

The authors declare that they have no competing interests.

## Authors’ contributions

YKL carried out most of the experiments, analyzed the data, and drafted the manuscript. DHJ assisted in the SPR sensor measurements. KSL and WMK designed and fabricated the WcBiM SPR sensor chips. YSS supervised the work and finalized the manuscript. All authors read and approved the final manuscript.
